# Long-Term Tillage and Crop Rotation Regimes Reshape Soil-Borne Oomycete Communities in Soybean, Corn, and Wheat Production Systems

**DOI:** 10.3390/plants12122338

**Published:** 2023-06-15

**Authors:** Alison Claire Gahagan, Yichao Shi, Devon Radford, Malcolm J. Morrison, Edward Gregorich, Stéphane Aris-Brosou, Wen Chen

**Affiliations:** 1 Ottawa Research and Development Centre, Agriculture and Agri-Food Canada, 960 Carling Ave., Ottawa, ON K1A 0C6, Canada; claire.gahagan@agr.gc.ca (A.C.G.); yichao.shi@agr.gc.ca (Y.S.); devon.radford@agr.gc.ca (D.R.); malcolm.morrison@agr.gc.ca (M.J.M.); ed.gregorich@agr.gc.ca (E.G.); 2 Department of Biology, University of Ottawa, 60 Marie Curie Prv., Ottawa, ON K1N 6N5, Canada

**Keywords:** crop rotation, metabarcoding, soil-borne oomycetes, soybean, tillage

## Abstract

Soil-borne oomycetes include devastating plant pathogens that cause substantial losses in the agricultural sector. To better manage this important group of pathogens, it is critical to understand how they respond to common agricultural practices, such as tillage and crop rotation. Here, a long-term field experiment was established using a split-plot design with tillage as the main plot factor (conventional tillage (CT) vs. no till (NT), two levels) and rotation as the subplot factor (monocultures of soybean, corn, or wheat, and corn–soybean–wheat rotation, four levels). Post-harvest soil oomycete communities were characterized over three consecutive years (2016–2018) by metabarcoding the Internal Transcribed Spacer 1 (ITS1) region. The community contained 292 amplicon sequence variants (ASVs) and was dominated by *Globisporangium* spp. (85.1% in abundance, 203 ASV) and *Pythium* spp. (10.4%, 51 ASV). NT decreased diversity and community compositional structure heterogeneity, while crop rotation only affected the community structure under CT. The interaction effects of tillage and rotation on most oomycetes species accentuated the complexity of managing these pathogens. Soil and crop health represented by soybean seedling vitality was lowest in soils under CT cultivating soybean or corn, while the grain yield of the three crops responded differently to tillage and crop rotation regimes.

## 1. Introduction

Oomycetes (or Oomycota) contain a group of fungal-like microorganisms within the kingdom Stramenopila, of which 60% of the species are considered pathogenic biotrophs, hemibiotrophs, or necrotrophs [1] and of great concern in agriculture [2], aquaculture [3], forestry, and natural ecosystems [4]. Depending on their host range, plant pathogens in oomycetes are considered generalist or specialist species, and such information often determines the control measures for pathogens in their respective categories. For example, *Globisporangium ultimum* is a pathogen with a wide host range that is associated with seedling damping-off disease complexes. As a generalist pathogen, *G. ultimum* is managed in plant production systems through fungicidal seed treatments specific to oomycetes [5]. Other oomycete pathogens are more limited in their host range, such as *Phytopthora* (*Ph.*) *sojae*, known only to be pathogenic on soybean (*Glycine max*) and lupins (*Lupinus*). Alongside chemical control measures, *Ph. sojae* in soybean is managed through single gene resistance pathways, or combinations of these genes to increase pathotype resistance [6]. The diversity of pathogen–host interactions and host specificity among soil-borne plant pathogenic oomycetes makes them a difficult group to manage. Moreover, soil-borne oomycetes are susceptible to changes in soil texture and organic matter, with a preference for cool, moist soils [7,8]. Studies have shown that agronomic practices, such as tillage and crop rotation, affect soil physicochemical properties and reshape the soil-borne microbiome (including the oomycetes community) structure, and can, consequently, be disruptive to soil health and fertility. Understanding how soil-borne oomycete communities respond to these common agronomic practices would help clarify best practices for regions with a high incidence of oomycete plant disease and provide the fundamental basis for establishing effective pest management and mitigation strategies for these important phytopathogens [9,10].

The use of tillage and crop rotation in managing soil-borne oomycetes may reduce the pathogen inocula or improve the soil’s natural capacity to suppress pathogenicity [11,12,13]. Conventional tillage (CT) usually involves fall moldboard plowing and spring cultivation and is practiced to reduce weed establishment [14] and soil compaction [15]. Practicing CT can lead to the loss of soil tilth, increased nutrient runoff, reduced soil quality, and disruption of the soil microbiome [16]. By contrast, no till (NT) promoted beneficial fungal and bacterial taxa compared to CT, under which the soils were enriched with plant pathogens, as reported by Srour et al. [17]. The diversity of bacterial taxa was also found to be lower in topsoil and higher in the deeper soil layers under CT compared to NT [18]. A greater microbial species diversity in soils under NT may lead to a more complex inter-species network, which may reinforce the suppression effects of beneficial microorganisms against the proliferation and growth of pathogenic species [19]. CT-mediated soil surface drying has been suggested as a management strategy since successful infection of the host by many oomycete species is dependent on zoospore mobility, supported by high soil water content [20].

Crop rotation, as a common agricultural practice, involves planting alternative crops sequentially on the same farmland for improved soil fertility and control of weeds, pests, and diseases. It has traditionally been encouraged to manage plant pathogens by mediating the availability of host plants from year to year [12,21,22]. Plant disease incidence and associated yields are impacted by the selection of crops in a cropping system and how they are rotated, with monoculture having reduced yields when compared to rotations involving other crops [23,24]. Crop rotation enriched plant growth-promoting bacterial [25] and disease-suppressive functional groups, such as those carrying the *prnD* gene that encodes the antifungal compound pyrrolnitrin [21]. Monoculture tomato soils were enriched in fungal genera containing potential pathogens, such as *Pseudogymnoascus*, *Fusarium*, and *Pyrenochaeta*, compared to soils under crop rotation [24].

Few studies have investigated the effects of tillage and crop rotation on soil-borne oomycetes at the community level. Srour et al. [17] showed that the beta diversity of oomycetes communities was significantly impacted by tillage but not fertilizer, and Blakney et al. [26] found that the oomycete community was sensitive to the cropping history of a site in *Brassicaceae* rotations, while the soil chemistry shaped the communities during dry years. Hwang, et al. [27] found that the levels of *Pythium* (*Py.*) inocula in monoculture soils, particularly peas, were greater than in rotation soils, which was reflected in disease incidence. *Pythium* and *Phytophthora* were the dominant genera recovered in a soybean–corn cropping system [28,29,30]. Oomycete pathogenicity on soybean, in particular, has been extensively studied and over 15 species of oomycetes, such as *Py. Aphanidenmatum*, *G. ultimum*, *G. irregulare*, and *G. cryptoirregulare*, have been shown to be pathogenic on soybean, although direct inoculation of soils with these recovered oomycete pathogens does not always reflect the severity of disease symptoms observed in soybean [28]. The relationship between oomycete plant pathogens and soybean seeds makes soybean an ideal initial crop to study disease symptoms in relation to oomycete species diversity and distribution in soils of varying rotation and tillage backgrounds [28,29,31,32,33,34,35,36,37,38]. The drive for maximizing the yields of high-value crops can often compete with the benefits of diversifying crop rotation systems. A thorough understanding of the potential disease ramifications due to rotation selection, especially within the oomycetes where little is known about the influence of crop rotation on community structure, may help to strengthen guidelines for more productive rotations.

In this study, we hypothesized that prolonged tillage and rotation practices can (1) exert a significant influence on the diversity and abundance of microbial communities, which may lead to a consistent increasing or decreasing trend over the course of the three-year study period, and (2) affect crop and soil health as represented by crop yield and seedling vitality of soybean. To test these hypotheses, a three-year study (2016–2018) at a long-term experimental site was carried out. The experimental field followed a split-plot design, incorporating two tillage levels (no till, NT, and conventional tillage, CT), with four randomly assigned rotations within each tillage treatment (Supplementary Figure S1). The rotations consisted of monocultures of dicot soybean (*Glycine max*, SSS), monocot corn (*Zea mays*, CCC), monocot wheat (*Triticum aestivum*, WWW), and a corn–soybean–wheat (CSW) rotation, which altered the available host range for oomycetes [39]. The post-harvest soil oomycete communities were characterized by analyzing the Internal Transcribed Spacer 1 (ITS1) region through metabarcoding. The objectives of this study were to examine the shifts in the soil-borne oomycete community in response to different combinations of tillage and crop rotation practices. By evaluating these dynamics, we aimed to provide valuable insights into better agricultural management practices for the effective management of soil-borne oomycetes, aligning with our hypotheses.

## 2. Results

A total of 3,148,276 high-quality reads were retained in the final amplicon sequencing variants (ASV) abundance table, with 30,272 ± 5785 (MEAN ± SD) reads per sample. In total, 292 ASVs (MEAN ± SD = 9 ± 3 per sample) were recovered from all samples.

### 2.1. Soil-Borne Oomycete Community Diversity and Compositional Structure

**Figure 1:**
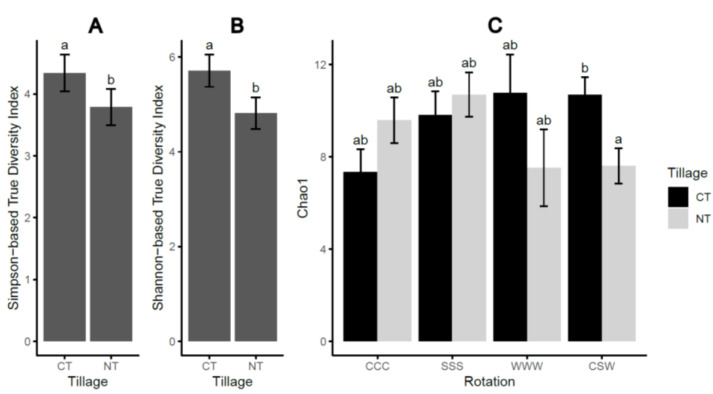
Alpha-diversity indices of the oomycetes communities in response to tillage (**A**,**B**) and the interaction of tillage and rotation (**C**). Chao1, Chao1 richness index; CT, conventional tillage; NT, no till; CCC, monoculture of corn; SSS, monoculture of soybean; WWW, monoculture of wheat; CSW, rotation of corn–soybean–wheat. Significant differences at α < 0.05 based on Tukey’s HSD test are indicated by different letters across the treatments. Error bars represent one standard error.

The tillage practices had a significant (*p* ≤ 0.05) impact on the alpha-diversity indices of the soil oomycete community. The results showed that under conventional tillage (CT), the Shannon-based true diversity index (Shannon-TD) and the Simpson-based true diversity index (Simpson-TD) were significantly higher compared to no-tillage (NT) practices (Table 1, Figure 1). This suggests that the increased diversity observed under CT was primarily driven by dominant species. Furthermore, the Chao1 index, which represents species richness, was significantly influenced (*p* = 0.001) by the interaction between tillage and rotation. Specifically, Chao1 values were higher under CT than NT when using crop rotation and CSW but not under monoculture practices. The CSW treatment resulted in increased Chao1 richness compared to continuous corn cropping (CCC) monoculture, but this effect was only observed under CT and not NT. These findings highlight the significance of rotation in influencing the abundance of less common species, as reflected in the Chao1 index. These results supported the first hypothesis that combined tillage and rotation practices significantly affected the soil-borne oomycetes community diversity. However, soil moisture content did not differ significantly between the tillage and rotation treatments (Supplementary Figure S2A). No significant correlations were observed between the soil moisture content and alpha-diversity indices (*p* > 0.05, Supplementary Figure S2B,D) or between the soil moisture content and the recovered oomycetes species (*p* > 0.05).

**Figure 2:**
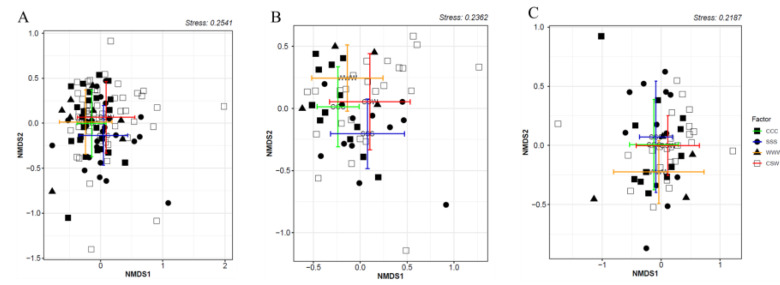
Soil oomycetes community structure in response to tillage (**A**) and rotation under CT (**B**) or under NT (**C**) using NMDS and PERMANOVA. CT, conventional tillage; NT, no till; CCC, monoculture of corn; SSS, monoculture of soybean; WWW, monoculture of wheat; CSW, rotation of corn–soybean–wheat. The central dots represent the means of the points on the two NMDS axes for the respective groups. The bars represent one standard deviation from the mean along both axes.

The results of the permutational multivariate analysis of variance (PERMANOVA) demonstrated significant effects of tillage (*F* = 4.83, *p* = 0.001; Figure 2A) and the interaction between tillage and rotation (*F* = 1.82, *p* = 0.01) on the structure of the soil-borne oomycetes community (Figure 2B,C). Rotation had a significant impact on the oomycete community structure under CT (*F* = 2.01, *p* = 0.003, Figure 2B), but not under NT (*F* = 1.20, *p* = 0.175; Figure 2C). The pairwise comparison and non-metric multidimensional scaling (NMDS) results revealed significant differences in the soil oomycete community structure under CT among the three monoculture treatments (*F* = 2.09, *p* < 0.001; Figure 2B). However, no significant differences were observed between the crops within the CSW rotation (*F* = 0.98, *p* = 0.468). Moreover, the PERMANOVA analysis indicated that soil moisture content had an insignificant impact on the beta diversity of the soil-borne oomycetes community (*p* > 0.05).

**Figure 3:**
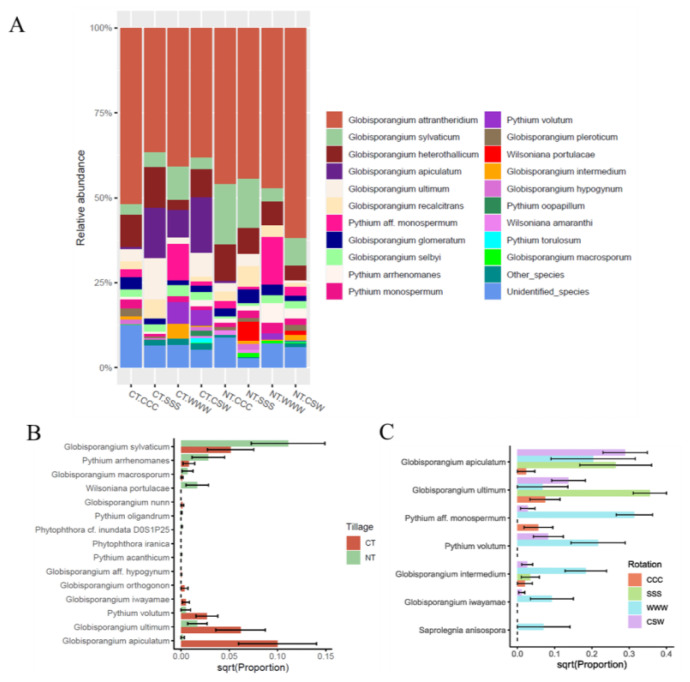
Abundance of oomycetes genera and species. (**A**) The 20 most abundant species under different tillage and rotation conditions. Other_species, identified species that were not among the 20 most abundant species; unidentified species, sequences that were not assigned to a known species. (**B**) Oomycetes species significantly affected by tillage (*p* < 0.05, linear mixed effect model) and species exclusively found under CT or NT. (**C**) Oomycetes species significantly affected by rotation under CT (*p* < 0.05). Error bars represent standard error. CT, conventional tillage; NT, no till; CCC, monoculture of corn; SSS, monoculture of soybean; WWW, monoculture of wheat; CSW, rotation of corn–soybean–wheat.

The oomycete ITS1 ASVs were classified into six (6) families, seven (7) genera, and 34 species. The dominant genera were *Globisporangium* (85.1%, 203 ASV), *Pythium* (10.4%, 51 ASV), and *Wilsoniana* (1.3%, 7 ASV). All other oomycete genera represented < 1% of the ASVs. The most abundant species belonged to the generalist genus *Globisporangium*, including *G. attrantheridium* (47.0%), *G. heterothallicum* (7.88%), *G. sylvaticum* (7.78%), *G. apiculatum* (5.92%), *and G. ultimum* (4.36%; Figure 3A). Phylogenetic analysis using the representative sequences of the ASVs confirmed the accuracy of the species-level classifications (Supplementary Figure S3).

Of the identified oomycete species, 22 were found under both CT and NT, 6 were exclusive to CT, and 6 were exclusive to NT (Table 2, Figure 3B). Tillage had a significant (*p* < 0.05) impact on eight species (Table 2). Abundances of *G. macrosporum*, *G. sylvaticum*, *Py. arrhenomanes*, and *Wilsoniana portulacae* were higher under NT compared to CT, while abundances of *G. iwayamae*, *G. ultimum*, *G. apiculatum*, and *Py. volutum* were higher under CT than NT (Figure 3B). Rotation also had a significant effect on seven species under CT, with increased abundances of *G. ultimum* under soybean monoculture, as well as *G. iwayamae*, *Pythium* sp. aff. monospermum, *Py. volutum*, and *Saprolegnia anisospore* under wheat monoculture (Table 2, Figure 3C). The abundance of *Pythium* sp. aff. monospermum was lower under CSW but higher (average of 10.6%) under wheat monoculture (Table 2). *G. iwayamae* showed a similar trend, with low abundance under CSW (relative abundance of 0.2%) and high abundance under wheat monoculture (relative abundance of 2.0%). In contrast, *S. anisospore* was abundant (2.0%) under wheat monoculture but was not detected under CSW (Table 2). Only *Py. volutum* was significantly (*p =* 0.004) affected by rotation under NT (Table 2).

**Figure 4:**
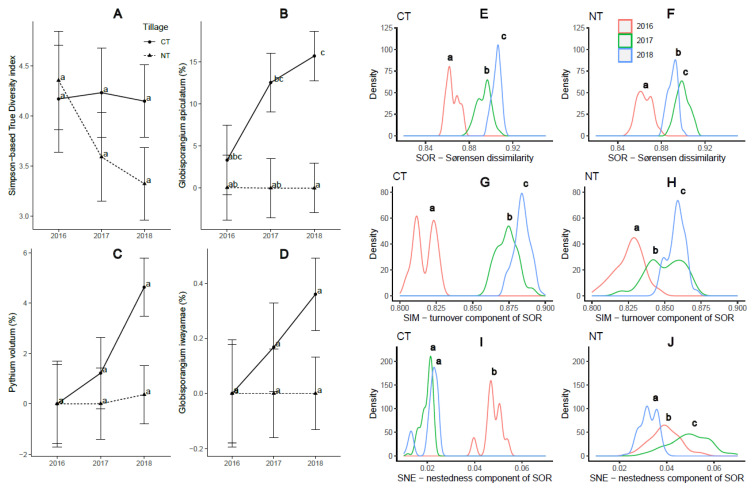
The impact of tillage practices on oomycetes community diversity and species abundance over the three-year study period (2016–2018). (**A**) The Simpson-based true diversity index (Simpson-TD) decreased under NT but not under CT. The relative abundance of (**B**) *Globisporangium apiculatum*, (**C**) *Pythium volutum*, and (**D**) *G. iwayamae* increased under CT but not under NT. (**E**–**J**) The impact of tillage practices on the beta diversity of oomycetes community: (**E**,**F**) the overall community dissimilarity (Sørensen dissimilarity, SOR) and (**G**,**H**) the turnover component of the Sørensen dissimilarity (Simpson dissimilarity, SIM) increased over the three growing seasons; (**I**,**J**) the nestedness component of the Sørensen dissimilarity (SNE) decreased over the three years under CT but not under NT. Different letters represent significant difference (*p* ≤ 0.05).

Since tillage practices had a greater impact on the oomycetes community compared to rotation patterns, we hypothesized that the implementation of long-term tillage regimes would result in a consistent trend of microbial abundance and diversity over the three-year study period. Our results supported this hypothesis, as alpha diversity represented by Simpson-TD showed a persistent but insignificant decrease under no tillage (NT) but not under conventional tillage (CT) (Figure 4A). This suggests that NT may have the potential to reduce the number of abundant oomycete species. Furthermore, a consistent increase in the abundance of *G. apiculatum*, *Py. volutum*, and *G. iwayamae* was observed under CT but not under NT, although these changes may not have reached statistical significance (Figure 4B–D). Interestingly, under CT, the overall dissimilarity of the oomycetes community (Sørensen dissimilarity, SOR) and the turnover component of the Sørensen dissimilarity (Simpson dissimilarity, SIM) increased over the three growing seasons, while the nestedness component of the Sørensen dissimilarity (SNE) showed the opposite trend (*p* ≤ 0.05; Figure 4E,G,I). Such a trend, however, was not observed under NT. Notably, the differences in SIM and SNE components between 2016 and the other two years were more than 2-fold greater under CT compared to NT (Figure 4F,H,J). These findings suggest that continuous CT practices may lead to decreased homogeneity of the soil oomycetes community.

### 2.2. Crop Yield and Soybean Seedling Emergence

Crop yield and seedling emergence tests were conducted to assess the soil and crop health under different tillage and crop rotation regimes. The growing seasons in 2016–2018 exhibited considerable variability in precipitation patterns (Figure S4). The analysis revealed that corn yield was significantly (*p =* 0.018) affected by tillage, with ~18% higher yields observed under NT compared to CT across the three-year period (Table 3). However, there was no significant effect of tillage on soybean and wheat yields (Table 3). In terms of crop rotation, a significant impact (*p* < 0.001) was observed on wheat yield, while no significant effect was found for corn and soybean yields. Wheat yields were ~78% higher under CSW compared to wheat monoculture (Table 3).

**Figure 5:**
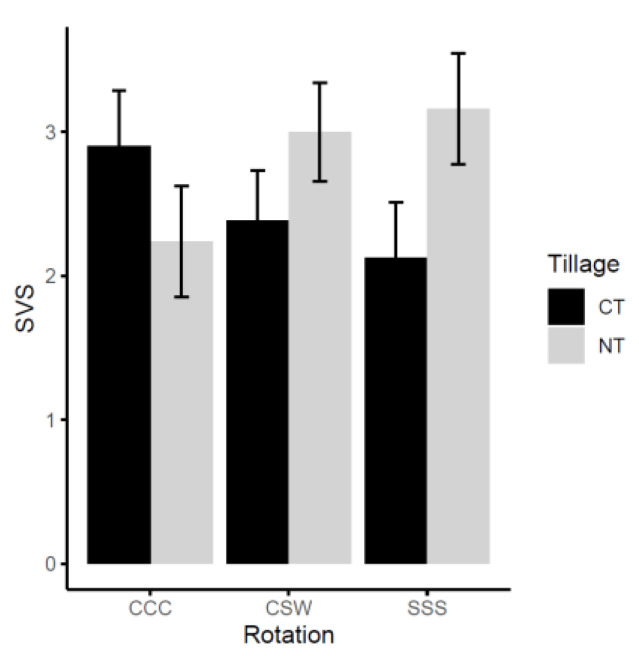
The impact of tillage and rotation on soybean seedling vitality score (SVS), with all pairwise comparisons showing no significant differences (*p* ≥ 0.05). CT, conventional tillage; NT, no till; CCC, corn monoculture; SSS, soybean monoculture; CSW, corn–soybean–wheat rotation.

When field soils collected during the study were planted with soybean in a growth cabinet under controlled conditions, seedling vitality (SVS; see Section 4.6 for methodology) was not significantly (*p* > 0.05) affected by tillage or rotation (Figure 5). Although not statistically significant, compared to CT, NT resulted in a decrease of approximately 30% in SVS under corn monoculture. In contrast, under soybean monoculture, NT led to a 21% increase in SVS, and under CSW rotation, it resulted in a 33% increase in SVS. Notably, among the treatments under corn monoculture, SVS was highest under CT and lowest under NT.

## 3. Discussion

In this study, the effects of long-term tillage and rotation regimes on the diversity and compositional structure of soil-borne oomycetes communities were evaluated. However, it is crucial to provide the environmental context in which this study was conducted. To achieve this, the crop yield as an indicator of soil and crop health was assessed, which helped us gain insights into the long-term effects of these agricultural practices on soil and crop conditions. We demonstrated that long-term tillage and rotation had a significant impact on crop yield, with variations observed between different crops, which is consistent with a previous study conducted at the same site by Morrison et al. [40]. In our study, crop rotation significantly increased wheat yield, particularly in 2016 and 2018, when the growing seasons were drier, but did not have a significant effect on corn and soybean yields. Morrison et al. [40] attributed the increase in wheat yield under rotation, 22% higher than that under monoculture, to the nitrogen-fixing soybean in the rotation. Similarly, Jalli et al. [41] observed increased wheat yield in more diverse cropping systems, with a 30% increase under NT and 13% under CT, along with reduced root disease after six years of rotation. These long-term observations highlight the value of crop rotation in enhancing crop productivity.

Oomycetes, despite their significance in crop production systems and their role in causing substantial crop yield losses, remain relatively understudied compared to bacteria and fungi. In our study, 34 oomycete species from 292 amplicon sequence variants (ASVs) were identified by analyzing the oomycetes ITS1 metabarcodes. The majority of oomycetes were attributed to two genera, *Globisporangium* and *Pythium*, which together accounted for 95% of the total oomycetes abundance. This finding is consistent with a previous study on rhizosphere-associated oomycetes of oak [42], which also reported *Globisporangium* and *Pythium* as the most abundant genera (>60%). Similarly, in soils imported to Norway attached to roots of ornamental trees and shrubs, *Pythium* (46%) was reported as the most abundant genus, followed by *Globisporangium* (6%) [43]. *Globisporangium* and *Pythium* species are largely considered generalists, capable of causing damping off and root rot in a wide range of agricultural crops, as indicated in Table 2 [44]. Recent taxonomic revision by Uzuhashi et al. [45] separated *Globisporangium* from *Pythium* as its own genus, which has led to the reclassification of several pathogens previously belonging to *Pythium* [43], such as the former *Py. sylvaticum* and *Py. ultimum* [45], now recognized as *G. sylvaticum* and *G. ultimum* [46], respectively. These species were highly abundant in the soils analyzed in our study. Zitnick-Anderson and Nelson [29] found that *G. attrantheridium*, *G. heterothallicum*, *G. hypogynum*, *G. intermedium*, and *G. irregulare*, caused pre-emergence damping off on soybean, resulting in less than 50% seedling emergence compared to 100% emergence in the control group. *G. heterothallicum*, in particular, was one of the most abundant oomycete species, particularly under corn and soybean monoculture. It has been reported as a pathogen affecting various crops, including corn [47] and soybean [33]. Studies focusing on oomycete pathogenicity on soybean have identified *G. heterothallicum* as the dominant species in North Dakota, representing 49% of the isolates [29]. However, Radmer et al. [28] reported that *G. heterothallicum* exhibited lower aggressiveness on soybean or corn, suggesting that its abundance could potentially influence the pathogenicity of more detrimental species through competition. Several species within the Pythiaceae family, such as *Py. arrhenomanes*, *Py. volutum*, *Py. oopapillum* and *Py. torulosum* have been implicated in causing diseases in the seeds and seedlings of soybean [29,32,39], corn [48,49], or wheat [50,51,52,53].

The impact of tillage practices, either alone or in conjunction with rotation regimes, was found to significantly influence the alpha and beta diversity of the oomycetes community in this study. Specifically, NT practices were associated with decreased alpha diversity, as indicated by the Simpson-TD and Shannon-TD indices (Figure 1). Furthermore, the Chao1 richness measure was also reduced under NT, particularly in the context of corn–soybean–wheat (CSW) rotation, but not in monoculture systems. The persistent downward trend in Simpson-TD over the three-year study period under NT suggests that this practice has the potential to reduce the abundance of oomycete species (Figure 4A). These findings contrast with those of Srour et al. [17] who reported no significant effect of tillage on Shannon’s diversity index of the oomycete community in soybean soils within a corn–soybean rotation. Interestingly, our results revealed an overall decrease in the homogeneity of the soil oomycete community under continuous conventional tillage (CT). This finding suggests that CT may not be an optimal practice for managing oomycete pathogens, as it disrupts the community structure and potentially favors the proliferation of certain species over others.

The observed decrease in alpha diversity of oomycetes under no till (NT) practices in this study could be linked to an increase in soil natural suppressiveness resulting from higher soil organic matter content. Previous research by Bongiorno et al. has suggested that reduced tillage practices have the potential to enhance soil suppressiveness through the presence of labile carbon and the positive influence on microbial biomass [11]. Additionally, a meta-analysis has indicated that NT tends to promote bacterial community diversity while having limited effects on fungal community diversity, possibly due to increased availability of labile carbon and improved water holding capacity in the soil [18]. The level of soil-borne pathogen suppression has also been linked to the quantity and quality of soil organic matter, as highlighted by Hoitink and Boehm [54]. Furthermore, studies have shown that the addition of compost, which enhances overall soil microbial activity, can improve the suppression of Pythium damping off, underscoring the importance of soil organic matter in soil suppressiveness against oomycetes [55].

The mobility and invasion ability of oomycete zoospores rely on the presence of adequate soil moisture [7,8,20]. Previous studies has demonstrated that reduced tillage coupled with crop rotation practices can enhance soil moisture and soil organic matter content by enhancing soil aggregation, promoting biological activities, and increasing water holding capacities [56,57]. However, in this study, we did not observe a direct connection between the tillage and rotation regimes and soil moisture content (Supplementary Figure S2). Additionally, soil moisture content did not exhibit significant associations with the alpha and beta diversity of the oomycete community or the abundance of individual oomycete species. This lack of association could be attributed to the sampling time during the fall, when the soil had already undergone compaction throughout the growing season, and when all plots experienced similar precipitation, temperature, and other climatic conditions.

The impact of conventional tillage (CT) on the oomycete community structure was significant, as observed in a previous study [17] which identified tillage as a primary factor influencing soil oomycete community heterogeneity. Among the 34 identified oomycete species, six were exclusively found under CT but absent under no till (NT), while another eight species showed the opposite trend (Table 2). This suggests that the response of oomycete species to tillage practices may vary depending on their ability to adapt to changes in environmental conditions. Similar findings were reported by Srour et al. [17] who exclusively recovered *G. attantheridium* only from NT plots (Figure 3B). Furthermore*, G. sylvaticum* and *Py. arrhenomanes*, confirmed pathogens of corn and soybean [33], were more abundant under NT than CT, aligning with previous research indicating increased abundance of *Pythium* spp. under reduced tillage [58,59]. Conversely, *G. apiculatum* was more abundant under CT and rarely present under NT, although this species is not a confirmed pathogen [60]. *G. ultimum*, another species with higher abundance under CT, particularly in soybean monoculture (Figure 3A), has been reported as a highly damaging pathogen for corn and soybean seeds and seedlings [32,33,38,61]. In addition, *Py. volutum*, highly abundant under CT, was present in lower abundance under NT and has been recognized as a significant pathogen in wheat [50]. Similarly, *G. apiculatum*, *Py. volutum*, and *G. iwayamae* exhibited consistent increases in abundance under CT over the three-year period, although these changes were not statistically significant (Figure 4B–D). Based on these observations, we hypothesize that soils under CT may have a reduced capacity to suppress oomycete pathogens compared to NT. To further investigate this phenomenon, our next step is to analyze the bacterial and fungal communities present in the same soil samples, which will provide additional insights into the potential interactions and dynamics between the soil microbiome and oomycete populations.

The abundance of certain *Pythium* species has been found to be correlated with various soil chemical properties, including pH, calcium and magnesium content, cation exchange capacity, and clay content [62,63]. Therefore, the shift in soil oomycete community composition observed in this study is likely associated with the alterations in soil physical and chemical properties caused by tillage practices. The presence of layered crop residue in no-till systems could potentially explain the occurrence of specific oomycete species, as it provides an ideal habitat for the buildup of primary inoculum [64,65]. Additionally, it is important to consider the timing of sampling, as it can influence the composition of oomycete communities. In this study, sampling was conducted in the fall, when a majority of the available plant tissue in the soil was dead, favoring saprophytic oomycetes such as *Pythium* and *Globisporangium*. Asad et al. [66] found that microbiome sampling early in the growing season was closely related to final seed quality. Therefore, it is likely that sampling oomycete communities in the spring or summer may result in different trends and provide further insights into their dynamics throughout the growing season.

In this study, the structure of the soil-borne oomycete community exhibited significant differences among various monoculture systems. Sapkota and Nicolaisen [67] has demonstrated that the crops grown prior to sampling can influence the composition of the oomycete community. In our study, high abundances of *G. apiculatum* and *G. ultimum* were found in soybean soils, while *Pythium* sp. aff. monospermum and *Py. volutum* were highly abundant in wheat soils. These findings confirm that different oomycete species exhibit preferences for specific crops as their hosts, indicating the influence of the crop itself on the oomycete community. Furthermore, the sensitivity of soil-borne oomycetes to crop rotation or cropping history was also observed by Blakney et al. [26], who found that the oomycete community displayed more distinct differences across different rotations compared to the bacterial community. The crop effect on the structure of the oomycete community could be attributed to various factors, including the influence of root exudates in the rhizosphere, the accumulation of crop-specific root pathogens or parasites, and the presence of plant-derived crop residues remaining in the soil after harvest [67].

Crop rotation has consistently proven to be an effective practice for reducing plant diseases caused by soil-borne pathogens [22,26,41,68,69,70], attributed to various factors, including alterations in soil physical properties and the presence of layers of crop residue on the soil surface, leading to changes in the structure and functions of the pathobiome community. In this study, rotation did not have a significant effect on the alpha diversity of the oomycete community, but it did significantly influence the beta diversity under CT. Specific oomycete species, such as *Pythium* sp. aff. monospermum*, G. iwayamae*, and *S. anisospore*, exhibited higher abundances in wheat monoculture but were present in low abundance or absent in wheat soils under the corn–soybean–wheat rotation. This suggests that rotation practices may reduce the levels of certain oomycete species by interrupting disease cycles. Similar observations were reported by Bargués-Ribera et al., where the inclusion of non-host crops led to a reduction in disease incidence [71]. The presence of *G. iwayamae* in various hosts, including wheat, has been documented in the USDA fungal database (https://nt.ars-grin.gov/fungaldatabases/) [72], and both *G. iwayamae* and *S. anisospore* are known to induce plant damping off or rot diseases. Our study highlights the benefits of crop rotation in reducing the abundance of oomycete plant pathogens, possibly due to the improved disease-suppressive capacity of soil microbiomes observed in more diverse rotations [21] and increased soil nitrogen levels because of the inclusion of soybean as a preceding crop, which ultimately contributed to higher wheat yields. No previous studies reported *Pythium* sp. aff. monospermum and *S. anisospore* in wheat soils. *Pythium* sp. aff. monospermum has been isolated from grapevine [73]*,* and *S. anisospore* is generally known as an aquatic pathogen [74]. The pathogenicity of these species in wheat soils is currently unknown. In this study, we did not observe a significant effect of rotation on the beta diversity of the soil oomycete community under NT. One possible explanation is that tillage and the resulting disruption of soil structure play a major role in driving the composition of the soil oomycete community in our study. The top 10 most abundant oomycete species under NT were not significantly influenced by rotation. Only *Py. volutum* showed a significant response to rotation, but this species is not highly abundant under NT and may have a limited contribution to the overall shift in the structure of the soil-borne oomycete community.

A considerable number of the identified oomycetes species are known pathogens associated with soybean (Table 2) [28,30]. To assess the overall health of the soils and determine if there is any association with the identified oomycete species, a greenhouse experiment was conducted to evaluate the vitality of soybean seedlings using the soils collected in 2016 and 2017. The aim was to examine the potential impact of tillage and rotation on seedling vitality scores (SVS). Neither tillage nor rotation showed a significant impact on SVS (Figure 5). There were also no significant associations between specific oomycete species and SVS. The reduced emergence observed in the NT-CCC, CT-SSS, and CT-CSW treatments could potentially be linked to the higher abundances of *G. heterothallicum* and *G. ultimum* (Table 2). These two species have been demonstrated to be highly pathogenic to soybean [28,30,75]. However, it is important to note that without isolation and/or molecular characterization [38,39], we cannot draw direct conclusions regarding the contribution of oomycetes to observed low SVS.

## 4. Materials and Methods

### 4.1. Study Site and Experimental Design

The soils were collected from a split-plot tillage-rotation experiment (Supplementary Figure S1) conducted at the Central Experimental Farm in Ottawa, ON, Canada (45°23′13.6″ N; 75°43′15.6″ W). This experiment was established in 1990. The soil at the site was a Matilda sandy loam (Melanic Brunisol, Canadian classification) with a pH (in CaCl_2_) of 6.8 [40]. The main plot factor consisted of two blocks, each measuring 89.1 m × 45.7 m. Within each block, half of the area was managed using no-tillage (NT) practices, while the other half was subjected to conventional tillage (CT). Within each main plot, there were subplots measuring 9.1 m × 45.7 m each, representing the rotation factor. Two replicates were assigned to each rotation pattern. This resulted in a total of four replicates for each combination of tillage and rotation treatment (Supplementary Figure S1).

For the CT plots, moldboard plowing (Overum DTL 5 Furrow plow, Västervik, Sweden) was performed in early November, followed by cultivation in the spring using a mulch finisher (John Deere 2310 Mulch Finisher, Augusta, Georgia) and a combination harrow (Kongskilde 2600 Triple K, Albertslund, Denmark) with rotatory baskets. The subplots were allocated to three crops: corn, soybean, or wheat. These crops were grown either in monoculture (CCC, SSS, CCC) or in two 3-year rotations (corn–soybean–wheat (CSW) or corn–wheat–soybean (CWS)). Each rotation pattern involved growing each crop in the rotation regime every year. This resulted in a total of nine subplot treatments (*n* = 3 for CSW, *n* = 3 for CWS, and *n* = 3 for monoculture). Within each main plot (representing the tillage effect), the subplots (representing crop x rotation) were duplicated in complete blocks. Hence, a total of 72 subplots were established, including randomized and duplicated subplots representing nine rotation sequences (*n* = 2 × 9) within each of the two duplicated main plots (*n* = 2 × 2) (Supplementary Figure S1).

The crop planting and management details were previously described by Morrison et al. [40]. In brief, wheat was planted in the first two weeks of May (450 seeds m^2^) using a Sunflower 9312 Multifunction Drill (Beloit, KS, USA) equipped with disc openers with a row spacing of 19 cm. Corn was seeded (7 seeds m^−2^) in the first two weeks of May with a John Deere 6-row corn planter set to the NT option; rows were 76 cm wide. Soybean was planted (55 seeds m^−2^) in the last two weeks of May with the Sunflower drill with 19 cm wide rows. Corn was fertilized with 224 kg N (as urea) ha^−1^ pre-plant broadcast and 40 kg ha^−1^ N–P_2_O_5_–K_2_O (18-18-18) at seeding. Wheat plots were fertilized with 100 kg N (as urea) ha^−1^ pre-planting. Soybean received no fertilizer. Pre-plant fertilizer was applied prior to spring tillage; therefore, it was integrated into the soil layer in CT plots but remained on the surface in the NT plots. Glyphosate was used to control weeds in commercial herbicide-resistant varieties of corn and soybean. Weeds were controlled in wheat with Buctril-M at 0.2 L ha^−1^ at the seedling stage.

Crop yields for soybean and wheat were measured by harvesting a central strip consisting of six rows from each plot using a plot combine (NurseryMaster, Wintersteiger, Germany). Corn yields were harvested using a John Deere combine (X9, John Deere, Moline, IL, USA). Grain yields were adjusted to a moisture content of 13% to ensure consistency. Corn crop residues were chopped down with a Loftness 180 flail-style chopper (Loftness, Hector, MN, USA), while the stubble of soybean and wheat was retained in the field without any further treatment. In CT plots, all crop residues were incorporated into the soil during the fall, while in NT plots, the residues remained on the soil surface. A weather station 700 m from the study site (45°22′57.34″ N, 75°42′50.96″ W) was used to collect precipitation and minimum and maximum daily temperature data throughout the growing seasons of 2016, 2017, and 2018.

### 4.2. Soil Sampling

In this study, the focus was on investigating the rotation effect, specifically within the corn–soybean–wheat rotation and monoculture plots (refer to Supplemental Figure S1). Soil sampling was carried out at the conclusion of the growing seasons from 2016 to 2018, following the harvest of each crop. The initial sampling in 2016 served as a proof of concept and included soils from soybean and corn monoculture plots, as well as CSW plots in which soybean was grown that year (24 plots). Subsequent sampling years expanded to incorporate additional rotation regimes: in 2017, CSW rotation plots in soybean and wheat, as well as corn (CCC) and soybean (SSS) monoculture treatments, were included (32 plots); in 2018, wheat monoculture (WWW) plots were added to the sampled plots (46 plots). Overall, a total of 102 soil samples were collected over the course of the three years. Given the unbalanced sampling strategy across the years, the analysis considered treatments that consisted of two tillage methods (CT and NT) and four rotations (CCC, SSS, WWW, and CSW). This approach allowed for a comprehensive investigation of the effects of tillage and rotation on the soil samples collected.

Soil sampling was conducted using a random staggered strategy, where soil cores were collected at a depth of 0–30 cm using a soil core sampler (Lamotte, Chesterton, MD, USA) with a diameter of 2 cm. In 2016 and 2017, a total of 50 soil cores were collected per plot, while in 2018, 15 soil cores were collected per plot. To ensure proper sanitation, all equipment was rinsed with distilled water, sterilized with 90% ethanol, and dried before moving on to sample different plots. The collected soil cores from each plot were combined and stored in a cold room at 4 °C until the sampling process was completed. Before sub-sampling, the soil samples were thoroughly mixed and passed through a 5 mm mesh to remove any rocks, plant material, and insects. A 15 mL sub-sample of the homogenized soil from each plot was then stored in a falcon tube and kept at −80 °C before downstream analysis.

Gravimetric soil moisture was measured by taking a 30 mL sample of soil at sampling for each plot, weighing it wet, and drying it at 60 °C until the weight was stable, approximately 7 days in this case. Dried soils were weighed, and soil moisture was calculated, as shown in Equation (1).

(1) $$Soil\;moisture\;content=\frac{wet\;weight-dry\;wieght}{wet\;weight}$$

### 4.3. DNA Extraction

The DNA was extracted using the FastDNATM Spin Kit for Soil (MP Biomedicals, Santa Ana, CA, USA) following the manufacturer’s instructions with the following modifications: a 2 mL microcentrifuge tube was used during the binding step rather than a 15 mL falcon tube, and a Percellys^®^ Evolution homogenizer (Bertin Instruments, Montigny-le-Bretonneux, France) was used instead of the MP Fast-Prep homogenizer. The soil was homogenized with silica and glass beads, then a column-binding step was used to remove debris where the DNA was bound to beads, and column elution was used to remove the protein and RNA. The purified DNA was then eluted into DNAse-free water. All samples were extracted in triplicate. The extracted DNA was stored at −25 °C.

The concentration of the DNA extract was measured using the Qubit dsDNA HS (High Sensitivity) Assay Kit on a Qubit Fluorometer (Thermo Fisher Scientific, Waltham, MA, USA). Three DNA replicates were pooled to 40 μL at a concentration of 10 ng μL^−1^ in 96-well PCR plates, which were stored at −25 °C. The plates containing the genomic DNAs were shipped overnight on dry ice to the Génome Québec Innovation Centre (Montreal, QC, Canada) for the preparation of sequencing libraries and amplicon-based metagenomics (or metabarcoding) sequencing using the Illumina MiSeq platform target fragment length was 100–300 bp with a target output of 15 Gb.

### 4.4. Sequencing Library Preparation and Illumina MiSeq Sequencing

At the Génome Québec Innovation Centre, the DNA libraries of the ITS1 region were prepared using SSU_ITS (5′-ACA CTG ACG ACA TGG TTC TAC ACG GAA GGA TCA TTA CCA CAC-3′) forward primer and the OOM_LO5.8S47c (5′-TAC GGT AGC AGA GAC TTG GTC TAT TAC GTA TCG CAG TTC GCA-3′) reverse primer (A. Levesque, personal communication). The initial PCR amplification was carried out in an 8 μL reaction volume comprised of 7 μL of the master mix (Supplementary Table S1) and 1 μL of sample DNA diluted to 1/50. The following thermocycling parameters were 15 min at 96 °C for initial melting, then 33 cycles through of 30 s at 96 °C, 30 s at 52 °C and 60 s at 72 °C, followed by a 10 min cool down at 72 °C. The amplicons were verified on a 2% agarose gel quantified and were purified using the sparQ PurMag Beads (Quantabio, Beverly, MA, USA). A secondary PCR was then performed to add the dual-indexed barcoding adapters. The PCR was carried out in a 7 μL reaction volume containing a 1/50 dilution of DNA to master mix. The PCR cycling parameters were: 15 min at 96 °C, 30 s at 96 °C, 30 s at 52 °C, 60 s at 72 °C and 10 min at 72 °C. The amplification was verified, and amplicons were purified as above. Indexation was performed with 1 μL of undiluted amplicon product secondary PCR. Indexed samples were verified on a 2% agarose gel and quantified using Quant-iT™ PicoGreen^®^ dsDNA Assay Kit (Life Technologies, Carlsbad, CA, USA). The sequencing library was made with an equal quantity in ng of DNA for each sample. Library DNA was cleaned with sparQ PureMag Beads. The library was quantified using Kapa Illumina GA with Revised Primers-SYBR Fast Universal Kit (Kapa Biosystems, Wilmington, MA, USA), and average fragment size was determined using the LabChip GX (PerkinElmer, Waltham, MA, USA). Before sequencing, 10% of the Phix control library was added to the amplicon pool for a final concentration of 8 pM. Sequencing was performed on the Illumina MiSeq platform with the MiSeq Reagent Kit v3 with 600 cycles. Sequencing was performed with LNA™ modified custom primers.

### 4.5. Metabarcoding Data Processing and Analysis

The Illumina MiSeq sequencing adapters were removed from the fastq files using Cutadapt ver.4.1 [76]. The paired-end raw reads were processed using DADA2 ver.1.14 [77] implemented in QIIME2 for denoising, chimera detection, and amplicon sequence variants (ASVs) inference using default parameters. The raw forward and reverse reads were truncated at 200 nt.

The taxonomic assignment was performed with an In-house complied reference database of oomycetes (denoted as oomycetes-ITS1-refDB) from GenBank. The oomycetes ITS sequences from GenBank were first downloaded in TinySeq XML format using the query “Oomycetes[Organism]” AND “150:2500[slen]” AND (“internal transcribed spacer 1” OR “ITS1”) NOT “sp.” NOT “uncultured” NOT “clone” NOT “whole genome” NOT” metagenome” (retrieved on 2 March 2022). In-house Perl and Bash scripts were developed to parse the XML file and to retrieve National Center for Biotechnology Information taxonomy for each sequence. The locations of rRNA gene regions (ITS1, 5.8S, ITS2, 28S) within each sequence were annotated by ITSx [78]. Only sequences, including the ITS1 region with sequence lengths between 150 and 500 bp, were retained. The final oomycetes-ITS1-refDB database contained 26,220 ITS1 reference sequences. This database can be downloaded from the bitbucket repository: https://bitbucket.org/wenchen_aafc/metabarcoding_oomycetes/downloads/, accessed on 12 June 2023.

The taxonomy assignments were initially classified via the q2-feature-classifer [79] implemented in QIIME2 against the oomycetes-ITS1-refDB database. Species-level identification of the oomycetes was improved by the Automated Oligonucleotide Design Pipeline (AODP), which identified all mutations distinguishing highly conserved DNA markers between close relatives [80,81]. The final taxonomy was improved by comparing and validating the results of three classifiers: AODP, q2-feature-classifier, and BLASTn at each taxonomic rank, in particular, at the species level. To assess the accuracy of the species-level assignments, the representative sequences of each ASV assigned to a specific species were combined with corresponding reference sequences of the species and its close relatives in oomycetes-ITS1-refDB. The combined sequence dataset was then aligned using MAFFT vers.7.407 [82], followed by the reconstruction of an approximate maximum likelihood (ML)_tree using FastTree (ver.2.1.0) with the –nt and –gtr options [83]. The ML tree was visualized in FigTree (ver.1.4.4, https://github.com/rambaut/figtree/releases, accessed on 12 June 2023).

### 4.6. Soybean Seedling Vitality Experiment

To evaluate the impact of tillage and rotation regimes on the potential pathogenicity of soil-borne microorganisms, including oomycetes, on soybean seedling emergence, seedling emergence tests were carried out under controlled growth cabinet conditions in 2016 and 2017. In this trial, 5 kg of soil were collected from selected subplots under different tillage × rotation treatments following the same procedure as the soil samples that were submitted for DNA extraction and sequencing. A total of 24 soil samples were collected in 2016 and 32 in 2017. The soils from each subplot were placed in a plastic tray (9 × 12 × 35 cm^3^). Thirty-two soybean seeds (variety: AC Mandor) were planted in a 4 column × 8 row grid at 2 cm deep in the soil. The growth cabinet was set at 15 °C with a 10 h photoperiod. Field capacity was calculated by filling a 2-inch plastic pot with field soil, saturating the soil, and weighing it after 16 h of draining. Soil moisture was maintained by weighing the trays and adjusting the water content to 80% of field capacity twice a day with distilled water. A final seedling vitality score was used to assess plant health at harvest: a score of “1” was given to seeds that did not germinate; “2” to seeds that germinated but had broken-off cotyledons and signs of rot on the stem and roots; “3” to seedlings that had emerged but had signs of necrosis on the cotyledons and delayed unifoliate emergence; “4” to seedlings with signs of necrosis on the stems and cotyledons as well as stunted and damaged unifoliates; “5” to seedlings with healthy unifoliates but signs of necrosis on the stems and cotyledons; and “6” to healthy seedlings (Supplementary Figure S5). To minimize edge effects, the seedlings of the end rows in proximity to the edge of the plug tray were not evaluated. The overall seedling vitality score (SVS) for each subplot was calculated using Equation (2), where *i* is the vitality score ranging from 1 to 6, *x_i_* is the number of seedlings with a given vitality score, and *n* is the total number of seedlings.

(2) $$\mathrm{SVS}=(\sum_{i=1}^{6}x_{i}\times i)/n$$

### 4.7. Statistical Analysis

All statistical analyses were performed in R (ver. 4.2.0) [84]. To avoid the risk of losing rare taxa (ASVs with low sequence counts), the ASV table was not rarefied but was Hellinger-transformed for multivariate analysis. The alpha-diversity indices are quantitative measures representing the diversity of ASVs in a sample. The Shannon Index (H), Simpson Index (D), and Chao1 index were calculated using vegan [85] and biodiversityR [86] packages. The Shannon-based true diversity (Shannon-TD = exp(H)) and the Simpson-based true diversity (Simpson-TD = 1/D) were calculated as suggested by Jost [87].

The alpha-diversity indices, crop yield, and SVS were checked for normality using the *shapiro.test* function and were transformed as needed. Linear mixed models were used to assess the main effects of tillage and rotation and their interaction on alpha-diversity indices, the relative abundances of species, and the SVS using the *lme* function in the nlme package [88] at a significance level of *p* ≤ 0.05. Tillage and rotation were treated as fixed effects and year as random effects. Linear mixed models were also used to assess the effect of rotation under each tillage treatment on the relative abundances of taxa at the species level, with rotation as a fixed effect and blocks and years as random effects. Linear mixed models were used to evaluate the tillage, rotation, and their interaction on corn, soybean, and wheat crop yields separately, with tillage and rotation as fixed effects, blocks as random effects, and year as repeated measurements.

The non-metric multidimensional scaling (NMDS) was performed to evaluate the overall impact of tillage and crop rotation on oomycete community heterogeneity using the *metaMDS* function in vegan [85]. The *adonis* function from the vegan package was used to perform permutational multivariate analysis of variance (PERMANOVA) [89] for determining the main effects of tillage, rotation, and the interaction effects, and also the effect of current crop under CSW treatment on the community heterogeneity of oomycete community based on the Bray–Curtis dissimilarity. Pairwise comparisons between treatments (tillage, rotation, or their combination) were conducted by using the *pairwise.perm.manova* function from the RVAideMemoire package [90] when one factor or interaction effect is significant. The community dissimilarity over the study years under CT and NT was evaluated by using the *beta.sample* function in the betapart package [91].

## 5. Conclusions

Our results supported the hypothesis that combined tillage and rotation regimes have significant impacts on the soil-borne oomycetes community and the overall health of the soil and crop. No-till (NT) practices can serve as a sustainable farming approach by effectively suppressing the soil oomycete community, while appropriate crop rotations under conventional tillage (CT) can influence soil health and oomycetes diversity. Different oomycete species responded differently to tillage and rotation practices, possibly due to variations in host availability and their unique adaptations to specific soil and environmental conditions. To fully harness the potential of tillage and rotation in managing soil-borne oomycete pathogens, future studies should investigate the impact of soil physicochemical properties on bacterial, fungal, and oomycete communities, construct cross-kingdom co-occurrence networks, and employ cultural and molecular diagnostic assays to facilitate the identification of causal agents responsible for observed diseases. These efforts will contribute to the development of more effective disease management strategies in agriculture.

## Figures and Tables

**Table 1:** The analysis of variance (ANOVA) *p*-values for the effects of tillage and rotation on alpha-diversity indices.

	DF	Simpson-TD ^1^	Shannon-TD ^2^	Chao1
Tillage (T)	1	0.014	0.007	0.160
Rotation (R)	3	0.613	0.563	0.4292
T × R	3	0.217	0.041	<0.001

^1^ Simpson-based true diversity index. ^2^ Shannon-based true diversity index.

**Table 2:** Relative abundance (%) of soil oomycete species affected by tillage (T), either conventional tillage (CT) or no tillage (NT), and rotation (R), including soybean monoculture (SSS), corn monoculture (CCC), wheat monoculture (WWW), and corn–soybean–wheat rotation (CSW). The table displays associated *p*-values from the analysis of variance, as well as potential hosts and disease symptoms. Species below 0.001% relative abundance are not included.

Species ^a^		Relative Abundance (%)								Analysis of Variance (*p*-Value) ^b^					Disease Note ^c^	Known Hosts ^c,d^
		CT				NT				T	R	T*R	R CT	R NT		
	No. ASV	CCC	SSS	WWW	CSW	CCC	SSS	WWW	CSW							
*Globisporangium aff. hypogynum*	1	0.000	0.000	0.000	0.153	0.000	0.000	0.000	0.000	0.320	0.78	0.772	0.7817	N/A		
*Globisporangium apiculatum*	20	0.664	16.294	7.970	16.522	0.000	0.000	0.000	0.002	**<0.001**	**0.036**	**0.031**	**0.041**	0.761		Grape
*Globisporangium attrantheridium*	49	57.290	33.493	40.079	41.743	38.209	46.590	47.258	59.105	0.230	0.448	**0.021**	0.066	0.138	Cavity spot lesions	*Daucus carota*, *Prunus*, **soybean**
*Globisporangium glomeratum*	11	2.273	2.341	1.381	2.454	1.770	2.658	3.176	2.208	0.96	0.833	0.936	0.988	0.734		**Soybean**
*Globisporangium heterothallicum*	27	10.438	11.844	2.942	6.979	13.878	7.603	7.139	3.802	0.539	**0.039**	0.558	N/A	0.076	Damping off	Pepper, **corn**, lentils, **soybean**, spinach, **wheat**
*Globisporangium hypogynum*	3	0.000	0.930	0.000	0.000	1.115	2.277	0.000	0.728	0.055	0.075	0.902	0.061	0.381	Root rots	**Soybean**
*Globisporangium intermedium*	3	1.000	0.000	4.290	0.388	0.000	0.995	0.281	1.700	0.755	0.074	**0.001**	**0.004**	0.407	Damping off, rots	Abutilon, antirrhinum, arabis, beet, begonia, carrot, cauliflower, **corn**, chamaecyparis, cherry laurel, chrysanthemum, cotoneaster, cucumber, erica, ferns, *Fragaria vesca*, godetia, hazel, hop, hyacinth, lettuce, leyland cypress, lupin, nasturtium, pea, pear, pelargonium, pepper, saintpaulia, **soybean**, strawberry, tomato, violet, yew
*Globisporangium irregulare*	2	0.000	0.000	0.000	0.247	0.074	0.000	0.000	0.000	0.559	0.867	0.563	0.782	0.347	Blight, damping off, root and other rots, etc.	**Soybean**, ** wheat**, **corn**
*Globisporangium iwayamae*	2	0.000	0.000	1.860	0.163	0.000	0.000	0.000	0.000	**0.024**	**0.001**	**0.0007**	**0.002**	N/A	Rots	Poaceae, **wheat**
*Globisporangium macrosporum*	2	0.000	0.000	0.000	0.181	0.055	1.220	0.797	0.546	**0.029**	0.658	0.738	0.782	0.682	Damping off, root rot	Iris, **corn**, **soybean**
*Globisporangium nunn*	1	0.000	0.000	0.000	0.000	1.042	0.000	0.000	0.000	0.333	0.349	0.339	N/A	0.3415		**Soybean**
*Globisporangium orthogonon*	1	0.000	1.578	0.000	0.000	0.000	0.000	0.000	0.000	0.305	0.323	0.264	0.3061	N/A		**Corn**
*Globisporangium pleroticum*	5	2.38	0.3683	0.000	0.193	0.952	1.170	0.000	1.760	0.7545	0.074	**0.009**	0.711	0.841		Lupins, peas, **soybean**
*Globisporangium recalcitrans*	14	1.103	6.190	0.000	1.321	3.613	4.969	3.326	1.693	0.131	0.194	0.566	0.219	0.435	Root rot, damping off	Beet, hebe
*Globisporangium rostratifingens*	5	0.000	0.000	0.000	0.329	0.725	0.233	0.000	0.000	0.699	0.671	0.107	0.529	0.174	Root rot	Pea, **soybean**, **corn**, ** wheat**
*Globisporangium selbyi*	8	2.200	2.223	3.130	2.350	0.591	0.774	2.410	2.290	0.702	0.859	0.996	0.943	0.750	Lesions	**Corn**, **soybean**
*Globisporangium sylvaticum*	25	4.970	2.344	9.556	3.796	19.620	12.984	3.839	7.282	**0.006**	0.330	0.086	0.334	0.063	Root disease, rots	Apples, carrot, cherry laurel, cress, chrysanthemum, cucumber, garlic, lettuce, onion, pea, radish, rhododendron, spinach, strawberry, yew, **wheat**
*Globisporangium ultimum*	11	2.346	14.656	1.834	6.589	2.490	2.820	0.000	1.348	**<0.001**	**0.008**	**0.011**	**0.002**	0.991	Blight, damping off, root rot, etc.	garlic, grape, hyacinth, lettuce, lily, lupin, melon, mustard, onion, parsley, pea, pear, pelargonium, pepper, poinsettia, primula, radish, rhododendron, rhubarb, **soybean**, spinach, strawberry, sweet pea, tomato, tulip, wallflower, yew
*Phytophthora* cf. *inundata D0S1P25*	1	0.000	0.000	0.000	0.000	0.000	0.237	0.000	0.000	0.180	0.093	0.152	N/A	0.092		
*Pythium acanthicum*	1	0.000	0.000	0.000	0.073	0.000	0.000	0.000	0.000	0.315	0.724	0.763	0.711	N/A	Downy mildew. Blight, damping off, rots, etc.	**Soybean**, **corn**
*Pythium aff. monospermum*	3	1.956	0.000	10.579	0.949	2.768	0.774	14.025	2.828	0.263	**<0.001**	0.794	**<0.001**	0.073		Grapevine
*Pythium arrhenomanes*	8	1.116	0.000	0.000	0.872	1.257	3.545	5.713	2.795	**0.014**	0.782	0.223	0.492	0.409	Blight, root rot	**Corn**, rice, barley, **wheat**
*Pythium monospermum*	12	2.109	0.872	1.683	1.187	1.274	1.928	3.108	2.005	0.367	0.857	0.725	0.476	0.962	Downy mildew. Root necrosis, not known as a strong pathogen	Cherry, juniper, spinach
*Pythium oligandrum*	1	0.000	0.000	0.000	0.000	0.000	0.000	0.000	0.200	0.338	0.763	0.772	N/A	0.762	Damping off; root, stem, and fruit rots	**Soybean**, ** wheat**
*Pythium oopapillum*	2	0.000	0.158	0.000	1.536	0.000	0.000	0.000	0.404	0.35	0.48	0.931	0.678	0.761	Root rot	**Soybean**
*Pythium torulosum*	1	0.000	0.000	0.000	1.326	0.000	0.000	0.000	0.000	0.144	0.503	0.477	0.469	N/A	Damping off, root rot	Pea, **soybean**, **corn**
*Pythium volutum*	6	0.000	0.000	6.287	4.399	0.000	0.002	1.895	0.053	**0.020**	**0.004**	0.143	**0.034**	**0.014**	Root rot	Barley, melon, morning glory, turfgrass, **wheat**
*Saprolegnia anisospora*	1	0.000	0.000	1.997	0.000	0.000	0.238	0.000	0.000	0.704	**0.024**	**0.01**	**0.006**	0.379		
*Saprolegnia torulosa*	1	0.000	0.175	0.000	0.000	0.000	0.000	0.000	1.093	0.142	0.384	0.181	0.306	0.265		
*Wilsoniana amaranthi*	1	0.000	0.000	0.000	0.638	1.252	0.743	0.000	0.000	0.646	0.876	0.369	0.782	0.515	White blister rust	Amaranth
*Wilsoniana portulacae*	6	0.000	0.000	0.000	0.000	0.000	5.157	0.000	1.132	**0.031**	0.19	0.211	N/A	N/A	White blister rust	Portulacaceae

^a^ Species are in alphabetic order. ^b^ *p*-values are in bold for significant impact of treatments. ^c^ Lifestyle and host information are acquired from https://nt.ars-grin.gov/fungaldatabases/ (accessed on 15 November 2022). ^d^ Hosts included in the study are in bold.

**Table 3:** The effects ^1^ of tillage (T) (conventional tillage; CT vs. no till; NT) and rotation (rotation vs. monoculture for each individual crop) on corn, soybean, and wheat yields (kg ha^−1^) during 2016–2018.

	Corn Yield	Soybean Yield	Wheat Yield
Tillage			
CT	9177 b	2257	1854
NT	10,806 a	2652	1795
Rotation			
Monoculture	9735	2404	1298 b
Rotation	10,248	2446	2305 a
Analysis of variance (*p*-values)			
Tillage (T)	**0.018**	0.226	0.548
Rotation (R)	0.442	0.577	**<0.001**
T × R	0.163	0.343	0.652

^1^ Different letters represent significant difference between treatments in tillage or rotation category at *p* = 0.05. Letter groups are not provided in the case of no significant differences.

## Data Availability

The raw paired-end metabarcoding sequencing data have been deposited in the Sequence Read Archive (SRA) under the BioProject accession PRJNA932837, Biosample accession SAMN33294720 to SAMN33294793.

## Supplementary Materials

The following supporting information can be downloaded at: https://www.mdpi.com/article/10.3390/plants12122338/s1, Figure S1: Field maps of the split-plot experimental design in each field sampling year. Figure S2: (A) The impact of tillage and rotation regimes on the soil moisture content, as measured by weighing soils at sampling, drying down the soils at 60 °C, and calculating gravimetric soil moisture content. The correlation relationships between the soil moisture content and the (B) Shannon-based true diversity index, (C) Simpson-based true diversity index, and (D) the chao1 index of the soil-borne oomycetes community; Figure S3: Validation of the classification accuracy of oomycetes ASVs at the species level using phylogenetic tree reconstruction. The 236 oomycetes ASVs (highlighted in red) were combined with reference sequences downloaded from the GenBank for multiple sequence alignment and approximate maximum likelihood tree reconstruction; Figure S4: Cumulative precipitation at the experimental site over the study years (2016–2018); Figure S5: Examples of soybean plants from each vitality score (1–6) rating where: 1, no germination; 2, broken-off cotyledons and signs of necrosis on roots; 3, seedlings with signs of necrosis on the cotyledons and stunted unifoliate emergence; 4, seedlings with signs of necrosis on the cotyledons and minimal stunting of the unifoliates; 5 seedlings with minimal cotyledon necrosis and healthy unifoliate development; 6, healthy seedlings with no signs of disease. Soybean plants were harvested after 21 days of growth in field soil at 15 °C constant temperature at 80% of saturated pot capacity. Scale bar = 1 cm.
