# Supplementary material for: Long-Term Tillage and Crop Rotation Regimes Reshape Soil-Borne Oomycete Communities in Soybean, Corn, and Wheat Production Systems
Source: Plants (Basel). 2023 Jun 15;12(12):2338. doi: 10.3390/plants12122338 (PMC10305274; doi:10.3390/plants12122338)
Supplement: Supplementary file 1 [file plants-2404544-supplementary.pdf › plants-2404544-supplementary.pdf]

2016

Plot #	Rotation	Current	Tillage	Plot #	Rotation	Current	Tillage
72	sss	S	NT	36	csw	C	CT
71	csw	W		35	cws	S	
70	csw	C		34	csw	W	
69	cws	S		33	sss	S	
68	www	W		32	cws	C	
67	ccc	C		31	www	W	
66	cws	W		30	cws	W	
65	csw	S		29	ccc	C	
64	cws	S		28	csw	S	
63	cws	W		27	www	W	
62	cws	C		26	csw	C	
61	sss	S		25	sss	S	
60	cws	S		24	csw	S	
59	www	W		23	cws	C	
58	csw	C		22	ccc	C	
57	csw	S		21	cws	W	
56	csw	W		20	csw	W	
55	ccc	C		19	cws	S	
54	sss	S	CT	18	ccc	C	NT
53	csw	S		17	csw	W	
52	cws	S		16	sss	S	
51	www	W		15	csw	S	
50	cws	C		14	csw	C	
49	cws	S		13	cws	C	
48	ccc	C		12	cws	W	
47	csw	W		11	www	W	
46	csw	C		10	cws	S	
45	csw	S		9	cws	S	
44	csw	W		8	csw	S	
43	cws	S		7	csw	C	
42	cws	W		6	cws	C	
41	csw	C		5	csw	W	
40	www	W		4	ccc	C	
39	ccc	C		3	sss	S	
38	sss	S		2	cws	W	
37	cws	C		1	www	W	

2017

Plot #	Rotation	Current	Tillage	Plot #	Rotation	Current	Tillage
72	sss	S	NT	36	csw	S	CT
71	csw	C		35	cws	C	
70	csw	S		34	csw	C	
69	cws	C		33	sss	S	
68	www	W		32	cws	W	
67	ccc	C		31	www	W	
66	cws	S		30	cws	S	
65	csw	W		29	ccc	C	
64	cws	C		28	csw	W	
63	cws	S		27	www	W	
62	cws	W		26	csw	S	
61	sss	S		25	sss	S	
60	cws	C		24	csw	W	
59	www	W		23	cws	W	
58	csw	S		22	ccc	C	
57	csw	W		21	cws	S	
56	csw	C		20	csw	C	
55	ccc	C		19	cws	C	
54	sss	S	CT	18	ccc	C	NT
53	csw	W		17	csw	C	
52	cws	C		16	sss	S	
51	www	W		15	csw	W	
50	cws	W		14	csw	S	
49	cws	C		13	cws	W	
48	ccc	C		12	cws	S	
47	csw	C		11	www	W	
46	csw	S		10	cws	C	
45	csw	W		9	cws	C	
44	csw	C		8	csw	W	
43	cws	C		7	csw	S	
42	cws	S		6	cws	W	
41	csw	S		5	csw	C	
40	www	W		4	ccc	C	
39	ccc	C		3	sss	S	
38	sss	S		2	cws	S	
37	cws	W		1	www	W	

2018

Plot #	Rotation	Current	Tillage	Plot #	Rotation	Current	Tillage
72	sss	S	NT	36	csw	W	CT
71	csw	S		35	cws	W	
70	csw	W		34	csw	S	
69	cws	W		33	sss	S	
68	www	W		32	cws	S	
67	ccc	C		31	www	W	
66	cws	C		30	cws	C	
65	csw	C		29	ccc	C	
64	cws	S		28	csw	C	
63	cws	C		27	www	W	
62	cws	S		26	csw	W	
61	sss	S		25	sss	S	
60	cws	W		24	csw	C	
59	www	W		23	cws	S	
58	csw	W		22	ccc	C	
57	csw	C		21	cws	C	
56	csw	S		20	csw	S	
55	ccc	C		19	cws	W	
54	sss	S	CT	18	ccc	C	NT
53	csw	C		17	csw	S	
52	cws	C		16	sss	S	
51	www	W		15	csw	C	
50	cws	S		14	csw	W	
49	cws	W		13	cws	S	
48	ccc	C		12	cws	C	
47	csw	S		11	www	W	
46	csw	W		10	cws	W	
45	csw	C		9	cws	W	
44	csw	S		8	csw	C	
43	cws	W		7	csw	W	
42	cws	C		6	cws	S	
41	csw	W		5	csw	S	
40	www	W		4	ccc	C	
39	ccc	C		3	sss	S	
38	sss	S		2	cws	C	
37	cws	S		1	www	W	

Supplementary Fig. S1. Field maps of the split-plot experimental design in each field sampling year (2016-2018), tillage levels (CT: conventional tillage, NT: no-till), rotation patterns (CCC: corn monoculture, SSS: soybean monoculture, WWW: wheat monoculture, CSW: corn-soybean-wheat rotation, CWS: corn-wheat-soybean rotation), as well as current crop (C: corn, S: soybean, W: wheat) are indicated for each field plot.

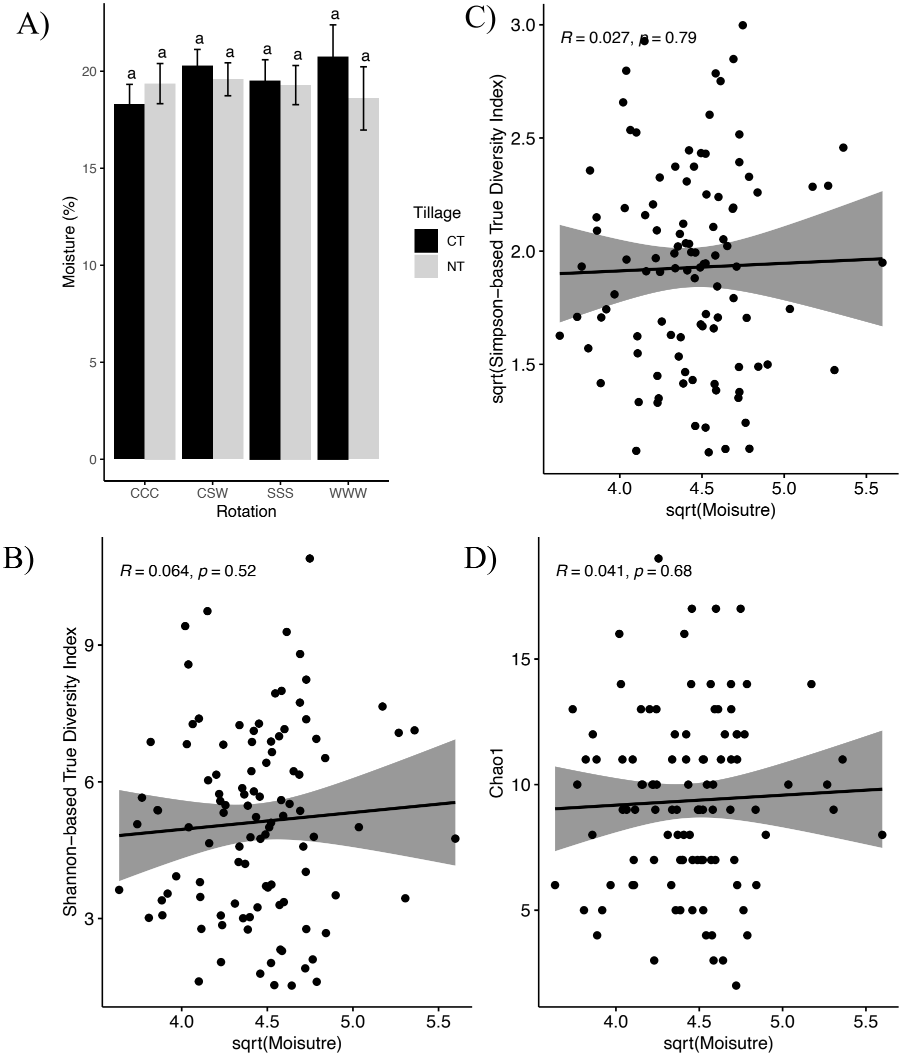

Supplementary Fig. S2: A) The impact of tillage and rotation regimes on the soil moisture content, as measured by weighing soils at sampling, drying down the soils at 60°C andcalculating gravometric soil moisture content. The correlation relationships between the soil moisture content and the B) Shannon-based true diversity index, C) Simpson-based true diversity index, and D) the chao1 index of the soil-borne oomycetes community.

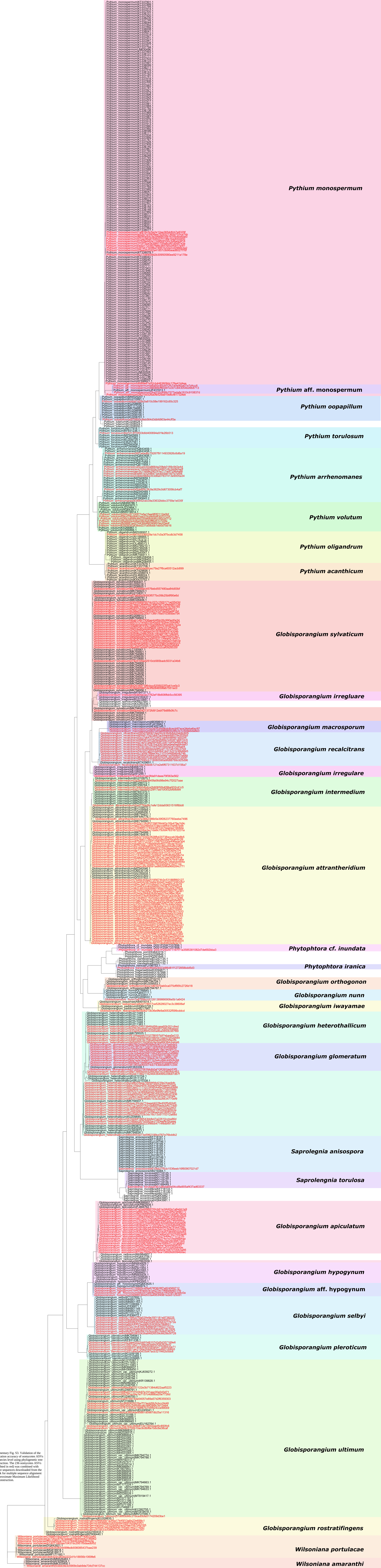

Supplementary Fig. S3. Validation of the classification accuracy of oomycetes ASVs at the species level using phylogenetic tree reconstruction. The 236 oomycetes ASVs (highlighted in red) was combined with reference sequences downloaded from the Genbank for multiple sequence alignment and Approximate Maximum Likelihood tree reconstruction.

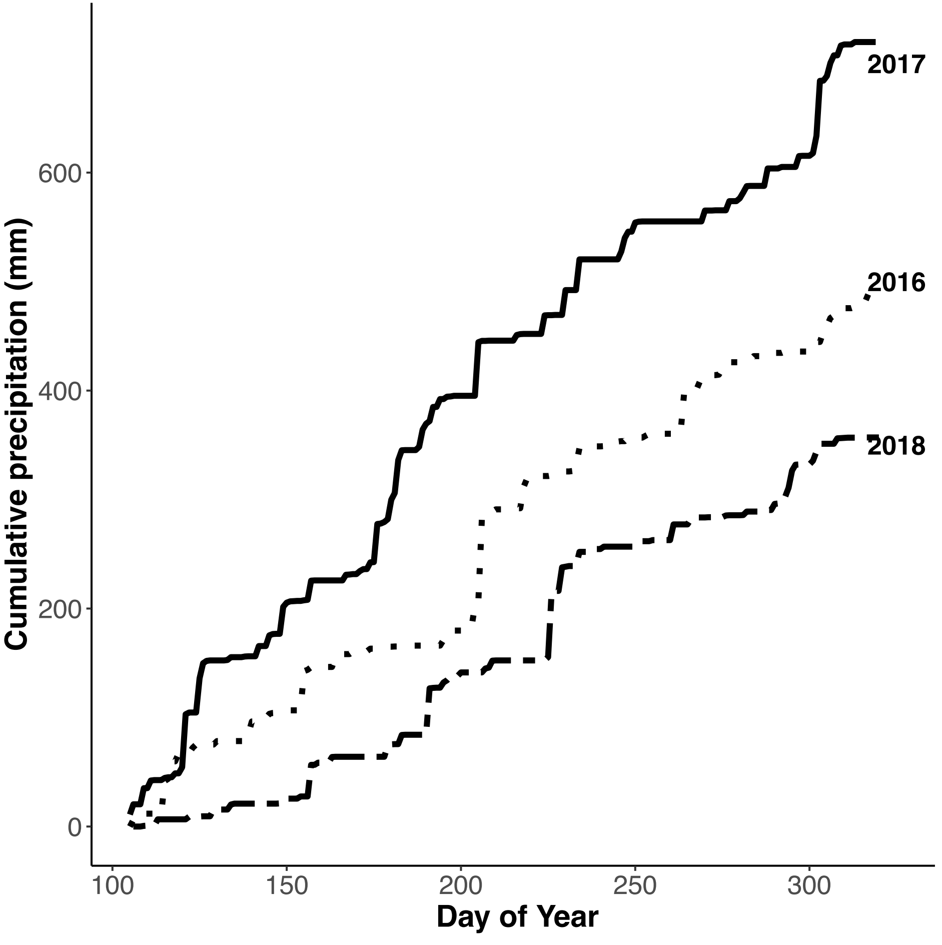

Supplementary Fig. S4. Cumulative precipitation at the field site over the study years (2016-2018).

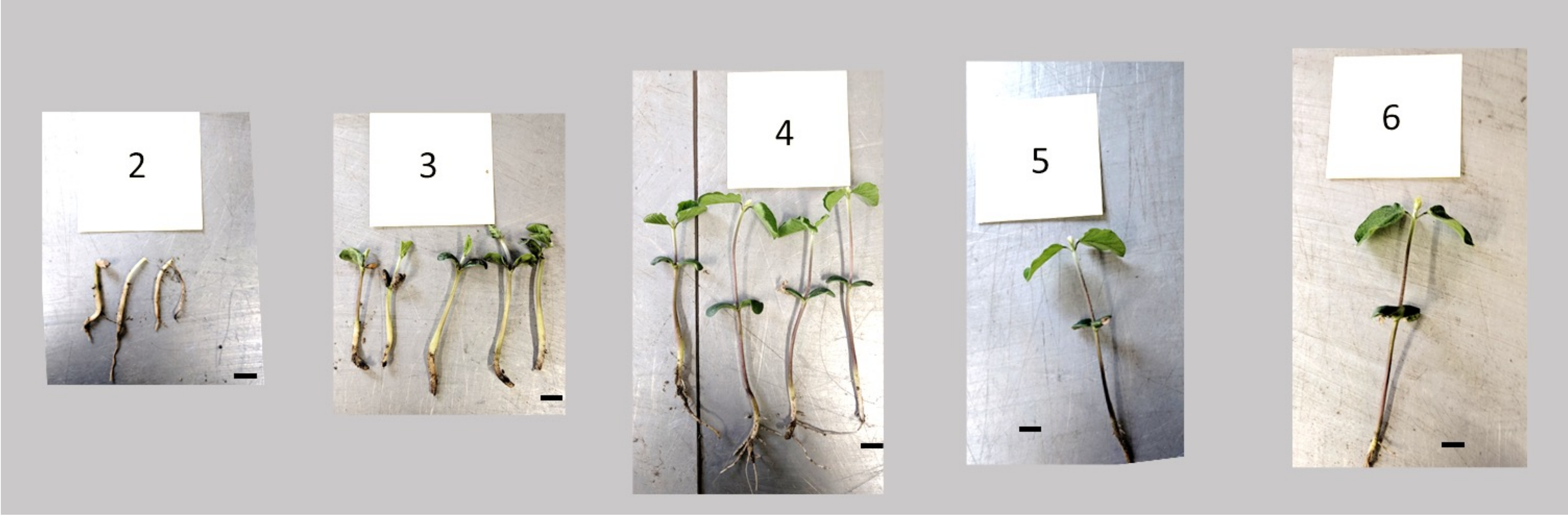

Supplementary Fig. S5. Examples of soybean plants from each vitality score (1-6) rating where: 1, no germination; 2, broken-off cotyledons and signs of necrosis on roots; 3, seedlings with signs of necrosis on the cotyledons and stunted unifoliate emergence; 4, seedlings with signs of necrosis on the cotyledons and minimal stunting of the unifoliates; 5 seedlings with minimal cotyledon necrosis and healthy unifoliate development; 6, healthy seedlings with no signs of disease. Soybean plants were harvested after 21 days of growth in field soil at 15 ^{o}C constant temperature at 80% of saturated pot capacity. Scale bar = 1 cm.
